# Trends in 5-year cancer survival disparities by race and ethnicity in the US between 2002–2006 and 2015–2019

**DOI:** 10.1038/s41598-024-73617-z

**Published:** 2024-09-30

**Authors:** Chongfa Chen, Lingdi Yin, Chunhui Lu, Guangfu Wang, Zhenyu Li, Feihu Sun, Huijuan Wang, Chenchen Li, Shangnan Dai, Nan Lv, Jishu Wei, Zipeng Lu, Feng Guo, Min Tu, Bin Xiao, Chunhua Xi, Kai Zhang, Qiang Li, Junli Wu, Wentao Gao, Xu Feng, Kuirong Jiang, Yi Miao

**Affiliations:** 1 Pancreas Center, Nanjing BenQ Medical Center, The Affiliated BenQ Hospital of Nanjing Medical University, Nanjing, China; 2 Pancreas Center, The First Affiliated Hospital of Nanjing Medical University, 300 Guangzhou Road, Nanjing, 210029 Jiangsu People’s Republic of China; 3 Pancreas Institute of Nanjing Medical University, Nanjing, China; 4 Vice-President, Pancreas Center, Pancreas Institute of Nanjing Medical University, The Affiliated BenQ Hospital of Nanjing Medical University, 300 Guangzhou Road, Nanjing, 210029 Jiangsu People’s Republic of China

**Keywords:** Trends, Relative survival rates, Cancer sites, Race/ethnicity, United States, Update, Computational biology and bioinformatics, Evolution, Gastroenterology, Health care, Medical research, Oncology, Pathogenesis, Risk factors, Mathematics and computing, Colorectal cancer, Pancreatic cancer, Breast cancer, Cancer epidemiology, Cancer prevention, Cancer screening

## Abstract

Racial and ethnic disparities persist in cancer survival rates across the United States, despite overall improvements. This comprehensive analysis examines trends in 5-year relative survival rates from 2002–2006 to 2015–2019 for major cancer types, elucidating differences among racial/ethnic groups to guide equitable healthcare strategies. Data from the SEER Program spanning 2000–2020 were analyzed, focusing on breast, colorectal, prostate, lung, pancreatic cancers, non-Hodgkin lymphoma, acute leukemia, and multiple myeloma. Age-standardized relative survival rates were calculated to assess racial (White, Black, American Indian/Alaska Native, Asian/Pacific Islander) and ethnic (Hispanic, Non-Hispanic) disparities, utilizing period analysis for recent estimates and excluding cases identified solely through autopsy or death certificates. While significant survival improvements were observed for most cancers, notable disparities persisted. Non-Hispanic Blacks exhibited the largest gain in breast cancer survival, with an increase of 5.2% points (from 77.6 to 82.8%); however, the survival rate remained lower than that of Non-Hispanic Whites (92.1%). Colorectal cancer survival declined overall (64.7–64.1%), marked by a 6.2% point drop for Non-Hispanic American Indian/Alaska Natives (66.3–60.1%). Prostate cancer survival declined across all races, with Non-Hispanic American Indian/Alaska Natives showing a decrease of 7.7% points (from 96.9 to 89.2%). Lung cancer, acute leukemia, and multiple myeloma showed notable increases across groups. Substantial racial/ethnic disparities in cancer survival underscore the notable need for tailored strategies ensuring equitable access to advanced treatments, particularly addressing significant trends in colorectal and pancreatic cancers among specific minority groups. Careful interpretation of statistical significance is warranted given the large dataset.

## Introduction

Cancer survival rates across racial and ethnic groups in the United States have long been a subject of intense scrutiny and concern. Numerous studies have consistently revealed disparities in survival outcomes among different populations, with African Americans, Hispanics, and certain other minority groups often exhibiting lower survival rates compared to non-Hispanic Whites for various cancer types^1–7^. These disparities persist despite advancements in cancer screening, treatment, and management over the past two decades^3^.

While improved survival has been observed for several cancers, such as breast cancer, non-Hodgkin’s lymphoma (NHL), and multiple myeloma, the extent to which these gains have been equally distributed across racial and ethnic lines remains unclear^8–10^.

This study aims to provide a comprehensive analysis of trends in 5-year relative survival rates for major cancer sites, including breast, colorectal, prostate, lung, pancreatic cancers, NHL, acute leukemia (AL), and multiple myeloma, across racial/ethnic groups in the United States^1^. By comparing data from two distinct periods, 2002–2006 and 2015–2019, we seek to elucidate whether the advancements in cancer treatment and management have translated into equitable improvements in survival outcomes for all populations.

Lung cancer has historically been associated with poor prognosis. In 2013, lung cancer screening guidelines were introduced in the United States, significantly impacting early-stage prognosis through the implementation of low-dose computed tomography (LDCT) screening^11^. Our study, by analyzing two distinct periods—2002–2006 and 2015–2019—provides a unique opportunity to assess survival outcomes both before and after the introduction of these guidelines. While the 2002–2006 cohort includes data up to 2012, prior to the implementation of the guidelines, the 2015–2019 cohort captures survival outcomes post-guideline. This comparison allows us to evaluate the differences in lung cancer survival before and after the introduction of the screening guidelines, with a particular focus on the disparities across racial and ethnic groups.

The selection of cancer types encompasses a diverse range, from those with established screening and treatment options (breast, colorectal, prostate) to cancers with limited screening and poor prognosis even at early stages (lung, pancreatic)^1^. Additionally, the inclusion of hematological malignancies like NHL, AL, and multiple myeloma, where long-term survival is possible even with late-stage diagnosis, provides a comprehensive perspective on survival trends across various cancer types^1^.

The data analyzed in this study were collected and categorized according to the revised race/ethnicity reporting guidelines, which were implemented to improve the accuracy and clarity of cancer statistics. This update aligns our analysis with the most current standards, ensuring that the disparities identified are reflective of these refined categorizations^11^.

By examining the intricate patterns and trends in cancer survival across different racial and ethnic groups, this study aims to highlight areas where healthcare policies and strategies may need to be adapted to ensure equitable cancer care and treatment outcomes for all segments of the population. The findings from this analysis have the potential to inform targeted interventions and resource allocation, ultimately working towards reducing the persistent disparities in cancer survival rates.

## Materials and methods

### Data source

The data for this study were derived from the Surveillance, Epidemiology, and End Results (SEER) Program of the National Cancer Institute, spanning a 20-year period from 2000 to 2020. The SEER 17 registries, which cover approximately 26.5% of the U.S. population based on the 2020 census, were utilized to ensure a comprehensive and representative dataset^12^. The study used de-identified data from the SEER database and was exempt from institutional review board approval.

### Cancer sites and populations

The analysis focused on major cancer sites, including breast, colorectal, prostate, lung, pancreatic cancers, non-Hodgkin lymphoma (NHL), acute leukemia (AL), and multiple myeloma. Due to the infrequency of individual leukemia types, AL data were aggregated for analysis. The study examined racial disparities among White, Black, American Indian/Alaska Native (AI/AN), and Asian/Pacific Islander (API) populations, as well as ethnic disparities between Hispanic and Non-Hispanic groups^1^.

### Exclusion criteria

Cases identified solely through autopsy or death certificates were excluded from the analysis to ensure data accuracy and reliability. Approximately 0.17–2.11% of patients were either classified into multiple racial/ethnic groups, lacked such information, or belonged to other unspecified groups. These patients were excluded from race/ethnicity-specific analyses to ensure data accuracy and reliability.

### Survival calculations

Age-standardized relative survival rates were calculated to enable comparisons across different demographic groups and time periods. The International Cancer Survival Standard (ICSS) was used for age standardization, employing the SEERStat software. Relative survival (RS) rates were computed to compare the survival of cancer patients with the expected survival of the general population^13–15^. The Ederer II method and life tables from the Centers for Disease Control and Prevention (CDC) were utilized to generate expected survival rates for the general population^16^.

### Period analysis

To provide the most recent survival estimates, the study employed period analysis instead of traditional cohort-based analysis. Period analysis focuses on the most recent survival experiences of patients, offering a more up-to-date representation of survival trends. For cases diagnosed between 2002 and 2015, direct calculations of 5-year RS rates were performed using the SEERStat software. For cases diagnosed between 2016 and 2019, period survival estimates were utilized due to the unavailability of complete 5-year follow-up data by 2020^17^.

### Statistical analysis

Patient counts, relative survival rates, standard errors, and associated p-values for trend analysis were extracted for each 5-year interval. The p-value threshold for statistical significance was set at 0.05. All analyses were conducted using Python 3.10.13 and SEERStat 3.8.2, incorporating custom macros for analyzing relative and period survival rates.

## Results

### Patient counts

Tables 1 and 2 provide detailed patient counts categorized by cancer type, race, and ethnicity. Across all subgroups, patient counts generally exceeded 100, except for acute leukemia among Non-Hispanic American Indian/Alaska Native individuals, where the count was relatively low. Across various cancer types, Non-Hispanic White patients consistently had the highest case counts, while Non-Hispanic American Indian/Alaska Native patients consistently had the lowest.

**Table 1:** Five-year relative survival (RS) for solid malignancies diagnosed in 2002–2006 and 2015–2019.

Cancer site/ethnicity	2002–2006			2015–2019			Difference	*P* value*
	*N*	5-year RS	SE	*N*	5-year RS	SE		
Breast cancer								
All	213,381	88.7	0.3	262,120	90.2	0.4	1.5	<0.00001
Non-Hispanic White	156,739	90.5	0.1	168,132	92.1	0.2	1.6	<0.00001
Non-Hispanic Black	20,279	77.6	0.5	28,543	82.8	0.6	5.2	<0.00001
Non-Hispanic American Indian/Alaska Native	1,043	84.2	2.4	1,641	87.2	2.3	3.0	<0.00001
Non-Hispanic Asian or Pacific Islander	15,106	89.0	0.5	27,335	90.1	0.5	1.1	<0.00001
Hispanic (all races)	20,214	86.2	0.5	36,469	87.7	0.5	1.5	<0.00001
Colorectal cancer								
All	146,933	64.7	0.4	143,754	64.1	0.6	− 0.6	<0.00001
Non-Hispanic White	106,612	66.0	0.2	90,161	65.3	0.3	− 0.7	<0.00001
Non-Hispanic Black	15,652	56.8	0.5	16,904	58.3	0.8	1.5	<0.00001
Non-Hispanic American Indian/Alaska Native	823	66.3	2.2	1,281	60.1	2.7	− 6.2	<0.00001
Non-Hispanic Asian or Pacific Islander	10,735	66.7	0.5	14,062	65.2	0.7	− 1.5	<0.00001
Hispanic (all races)	13,111	61.6	0.5	21,346	62.8	0.7	1.2	<0.00001
Prostate cancer								
All	236,194	98.3	0.2	231,346	95.7	0.4	− 2.6	<0.00001
Non-Hispanic White	171,549	99.1	0.1	153,668	96.6	0.2	− 2.5	<0.00001
Non-Hispanic Black	31,110	96.1	0.3	37,852	94.9	0.5	− 1.2	<0.00001
Non-Hispanic American Indian/Alaska Native	859	96.9	1.4	974	89.2	3.0	− 7.7	<0.00001
Non-Hispanic Asian or Pacific Islander	11,505	96.6	0.4	13,235	93.1	0.6	− 3.5	<0.00001
Hispanic (all races)	21,171	95.7	0.3	25,617	93.0	0.5	− 2.7	<0.00001
Lung cancer								
All	182,055	16.1	0.2	176,120	24.7	0.4	8.6	<0.00001
Non-Hispanic White	143,148	16.5	0.1	128,503	24.8	0.2	8.3	<0.00001
Non-Hispanic Black	18,459	12.5	0.3	19,171	21.5	0.5	9.0	<0.00001
Non-Hispanic American Indian/Alaska Native	863	12.4	1.2	1,114	20.6	2.1	8.2	<0.00001
Non-Hispanic Asian or Pacific Islander	9,903	17.6	0.4	14,394	28.6	0.7	11.0	<0.00001
Hispanic (all races)	9,682	15.7	0.4	12,938	24.4	0.7	8.7	<0.00001
Pancreatic cancer								
All	34,431	6.3	0.3	48,860	12.4	0.5	6.1	<0.00001
Non-Hispanic White	24,712	6.3	0.2	31,852	12.5	0.3	6.2	<0.00001
Non-Hispanic Black	3,892	5.0	0.4	5,718	10.5	0.7	5.5	<0.00001
Non-Hispanic American Indian/Alaska Native	195	6.3	1.8	316	3.6	0.9	− 2.7	<0.00001
Non-Hispanic Asian or Pacific Islander	2,299	8.0	0.6	4,313	14.9	0.9	6.9	<0.00001
Hispanic (all races)	3,333	6.8	0.5	6,661	12.5	0.7	5.7	<0.00001

This table compares the 5-year relative survival rates for patients diagnosed with solid malignancies (breast, colorectal, prostate, lung, and pancreatic cancers) between two periods: 2002–2006 and 2015–2019. The table is divided into sections for each cancer type, further broken down by racial and ethnic groups: All Patients, Non-Hispanic White, Non-Hispanic Black, Non-Hispanic American Indian/Alaska Native, Non-Hispanic Asian or Pacific Islander, and Hispanic (all races). For each group, the number of patients, 5-year RS percentages, and the standard error (SE) are provided for both periods. The table also includes the statistical significance (P-value) of the change in survival rates between the two periods, testing against the null hypothesis of no change in survival. Significance of the change in 5-year RS between the two periods, with a P-value < 0.00001 indicating a statistically significant change. *RS* Relative survival, *SE* Standard error.

**Table 2:** Five-year relative survival rates for common hematological malignancies diagnosed in 2002–2006 and 2015–2019.

Cancer site/ethnicity	2002–2006			2015–2019			Difference	*P* value*
	*N*	5-year RS	SE	*N*	5-year RS	SE		
Non-Hodgkin’s lymphoma								
All	59,646	66.2	0.6	68,771	72.2	0.8	6.0	<0.00001
Non-Hispanic White	44,779	68.5	0.3	46,014	75.0	0.4	6.5	<0.00001
Non-Hispanic Black	4,417	57.6	1.2	5,403	66.9	1.4	9.3	<0.00001
Non-Hispanic American Indian/Alaska Native	263	63.7	4.2	387	64.1	4.8	0.4	0.215798
Non-Hispanic Asian or Pacific Islander	3,617	60.8	0.9	5,954	65.8	1.1	5.0	<0.00001
Hispanic (all races)	6,570	59.8	0.8	11,013	67.1	0.9	7.3	<0.00001
Acute leukemia								
All	13,990	17.9	0.7	16,096	26.5	1.1	8.6	<0.00001
Non-Hispanic White	9,683	18.1	0.4	9,631	27.6	0.7	9.5	<0.00001
Non-Hispanic Black	1,037	15.3	1.1	1,396	21.0	1.6	5.7	<0.00001
Non-Hispanic American Indian/Alaska Native	–	–	–	121	7.1	1.3	–	–
Non-Hispanic Asian or Pacific Islander	1,074	19.7	1.2	1,625	26.7	1.7	7.0	<0.00001
Hispanic (all races)	2,196	17.4	0.9	3,444	25.5	1.3	8.1	<0.00001
Myeloma								
All	18,159	42.5	0.9	27,032	58.2	1.2	15.7	<0.00001
Non-Hispanic White	11,843	43.8	0.5	15,683	58.6	0.7	14.8	<0.00001
Non-Hispanic Black	3,304	41.0	1.0	5,464	59.2	1.3	18.2	<0.00001
Non-Hispanic American Indian/Alaska Native	109	42.1	5.5	202	50.4	6.7	8.3	<0.00001
Non-Hispanic Asian or Pacific Islander	931	39.8	1.7	1,752	58.5	1.9	18.7	<0.00001
Hispanic (all races)	1,972	38.9	1.2	3,931	55.6	1.4	16.7	<0.00001

This table presents a comprehensive analysis of the 5-year relative survival (RS) rates for patients diagnosed with common hematological malignancies, including acute leukemia, myeloma, and non-Hodgkin lymphoma (NHL), across two distinct time periods: 2002–2006 and 2015–2019. The data is categorized into subgroups based on race and ethnicity, encompassing All Patients, Non-Hispanic White, Non-Hispanic Black, Non-Hispanic American Indian/Alaska Native, Non-Hispanic Asian or Pacific Islander, and Hispanic (all races). Each subgroup details the number of patients, the 5-year RS percentages, and the standard error (SE) for both time frames. The table highlights the statistical significance of changes in survival rates over these periods, with a P-value < 0.00001 indicating a highly significant change, except for Non-Hispanic American Indian/Alaska Native in NHL, where a different P-value is noted. *RS* Relative survival, *SE* Standard error.

### Breast cancer

From 2002 to 2019, significant improvements were observed in 5-year relative survival (RS) rates for breast cancer across all racial/ethnic groups. Overall, the rate increased by 1.5% points, rising from 88.7 to 90.2% (*P* < 1 × 10⁻^5^). Non-Hispanic Black patients experienced the largest gain, with rates rising from 77.6 to 82.8%, a 5.2% point increase. Non-Hispanic Whites saw an increase of 1.6% points (from 90.5 to 92.1%), Non-Hispanic Asians/Pacific Islanders had an increase of 1.1% points (from 89.0 to 90.1%), Non-Hispanic American Indians/Alaska Natives experienced a 3.0% point increase (from 84.2 to 87.2%), and Hispanics had a 1.5% point increase (from 86.2 to 87.7%). Non-Hispanic Black patients showed improvement but had lower survival rates compared to Non-Hispanic Whites.

### Prostate cancer

Overall, 5-year RS rates for colorectal cancer showed a slight decline of 0.6% points, from 64.7 to 64.1% (*P* < 1 × 10⁻^5^). Non-Hispanic Whites experienced a 2.5% point decrease (99.1–96.6%), while the decline was smaller for Non-Hispanic Blacks (96.1–94.9%, 1.2% points). Non-Hispanic American Indians/Alaska Natives experienced a 7.7% point decrease (96.9–89.2%). Non-Hispanic Asians/Pacific Islanders saw a 3.5% point decline (96.6–93.1%), and Hispanics had a 2.7% point decrease (95.7–93.0%).

### Colorectal cancer

The overall survival for colorectal cancer declined slightly (64.7–64.1%), with the most notable decrease seen among Non-Hispanic American Indian/Alaska Native individuals, where survival dropped by 6.2% points (66.3–60.1%). This sharp decline underscores the unique challenges faced by this population. Non-Hispanic Whites experienced a 0.7% point decrease (66.0–65.3%), while Non-Hispanic Blacks showed a 1.5% point increase (56.8–58.3%), which is notable given that survival rates declined for most other racial/ethnic groups during the same period. The disparity remains in the absolute survival percentages, with Non-Hispanic Blacks still having lower survival (58.3%) compared to Non-Hispanic Whites (65.3%). Non-Hispanic Asians/Pacific Islanders also saw a 1.5% point decrease (66.7–65.2%), while Hispanics had a 1.2% point increase (61.6–62.8%).

### Pancreatic cancer

The survival rate for pancreatic cancer increased by 6.1% points, from 6.3 to 12.4% overall (*P* < 1 × 10⁻^5^). Among non-Hispanic Whites, survival rates rose from 6.3 to 12.5%, reflecting an improvement of 6.2% points (*P* < 1 × 10⁻^5^). Non-Hispanic Blacks experienced a rise in survival rates from 5.0 to 10.5%, an increase of 5.5% points (*P* < 1 × 10⁻^5^). Non-Hispanic Asians or Pacific Islanders showed an increase from 8.0 to 14.9%, a 6.9% point increase (*P* < 1 × 10⁻^5^). Hispanics (All Races) saw an enhancement in survival rates from 6.8 to 12.5%, an increase of 5.7% points (*P* < 1 × 10⁻^5^). However, non-Hispanic American Indians/Alaska Natives were the only group to exhibit a decline, with survival rates decreasing from 6.3 to 3.6%, a reduction of 2.7% points (*P* < 1 × 10⁻^5^).

### Lung cancer

Substantial improvements were noted in 5-year RS rates for lung cancer from 2002 to 2019, with an overall increase of 8.6% points, rising from 16.1 to 24.7% (*P* < 1 × 10⁻^5^). Non-Hispanic Asians/Pacific Islanders had the largest gain of 11.0% points (17.6–28.6%), followed by Non-Hispanic Blacks (12.5–21.5%, 9.0% point increase), Non-Hispanic Whites (16.5–24.8%, 8.3% point increase), Hispanics (15.7–24.4%, 8.7% point increase), and Non-Hispanic American Indians/Alaska Natives (12.4–20.6%, 8.2% point increase).

### Non-hodgkin lymphoma

From 2002 to 2019, 5-year relative survival rates for non-Hodgkin lymphoma (NHL) increased across all racial/ethnic groups, with an overall rise of 6.0% points, from 66.2 to 72.2% (*P* < 0.00001). While Non-Hispanic Blacks exhibited a observable improvement in survival rates from 57.6 to 66.9%, a 9.3% point gain (*P* < 0.00001), it is important to note that other groups also had lower survival rates. Non-Hispanic American Indian/Alaska Native (AI/AN) individuals showed only a modest increase of 0.4% points, from 63.7 to 64.1% (*P* = 0.215798). Non-Hispanic Asian or Pacific Islander (API) patients experienced a 5.0% point increase, from 60.8 to 65.8% (*P* < 0.00001). Additionally, Hispanics showed a 7.3% point increase, from 59.8 to 67.1% (*P* < 0.00001), with only a 0.2% difference compared to Non-Hispanic Whites (75.0%).

### Multiple myeloma

Notable increases in 5-year RS rates for multiple myeloma were observed across all racial/ethnic groups from 2002 to 2006 to 2015–2019. Non-Hispanic Asians/Pacific Islanders saw the largest gain, with an 18.7% point increase (from 39.8 to 58.5%), followed closely by Non-Hispanic Blacks, who experienced an 18.2% point increase (from 41.0 to 59.2%). Improvements were also observed in Non-Hispanic Whites, with a 14.8% point increase (from 43.8 to 58.6%), and Hispanics, with a 16.7% point increase (from 38.9 to 55.6%). Non-Hispanic American Indians/Alaska Natives showed a comparatively modest increase of 8.3% points (from 42.1 to 50.4%).

### Acute leukemia

Overall, 5-year RS rates for acute leukemia increased significantly by 8.6% points, from 17.9 to 26.5% (*P* < 1 × 10⁻^5^) between 2002 and 2019. Non-Hispanic Whites saw an increase from 18.1 to 27.6%, a 9.5% point increase, followed by Hispanics (17.4–25.5%, 8.1% point increase), Non-Hispanic Asians/Pacific Islanders (19.7–26.7%, 7.0% point increase), and Non-Hispanic Blacks (15.3–21.0%, 5.7% point increase). Data for Non-Hispanic American Indians/Alaska Natives was lacking from 2002 to 2006, but their 2015–2019 survival rate was 7.1%.

## Discussion

### Persistent racial and ethnic disparities in cancer survival: a multifaceted challenge

Our comprehensive analysis provides insights into an intricate landscape of survival disparities across various cancers between 2002 and 2006 and 2015–2019, highlighting persistent inequities among different racial and ethnic groups in the United States. Despite overall improvements in survival rates for most cancers, these advancements have not been uniformly experienced, underscoring an ongoing challenge in achieving healthcare equity^2,7,18^. In this discussion, we will systematically address the findings by cancer type, maintaining coherence with the structure of the Results section.

### Breast and prostate cancer: contrasting trajectories

The improvement in breast cancer survival across all groups, including Non-Hispanic Black patients, was noted^19^. However, the persistently lower survival rates in this group compared to Non-Hispanic Whites highlight the need for continued efforts to bridge this gap^20^. In contrast, the decline in prostate cancer survival across all races, with Non-Hispanic American Indian/Alaska Native patients showing the most pronounced decrease^21^.

### Colorectal cancer: emerging trends and enduring racial divides

Our investigation into colorectal cancer survival rates reveals a nuanced and complex picture. The marginal decline in overall survival from 2002 to 2006 to 2015–2019 deviates from global progress in disease management, potentially reflecting deficiencies in screening methodologies or treatment protocols employed nationwide^22–24^. Interestingly, this downward trend contrasts with the notable improvements among Non-Hispanic Black patients. However, a disparity persists when compared to Non-Hispanic White survival rates, highlighting an ongoing challenge in bridging racial divides in healthcare outcomes^25^. The overall decrease, along with these disparities, indicates the need to examine the healthcare system’s ability to address the needs of diverse racial and ethnic groups^2^.

### Pancreatic cancer: a pressing concern for non-hispanic American Indian/Alaska native populations

Conversely, our pancreatic cancer findings reveal a more positive overall trend, with increased survival rates suggesting advancements in early detection and treatment approaches^26^. Despite overall improvements in pancreatic cancer survival, the significant decline among Non-Hispanic American Indian/Alaska Native populations remains a cause for concern^27^. This could involve broadening access to early detection, advancing treatment options, and concerted efforts to overcome systemic barriers to quality healthcare access for these communities^28,29^.

### Lung cancer, NHL, and myeloma: advances and lingering disparities

The substantial improvements in lung cancer and non-Hodgkin lymphoma survival across all groups reflect treatment advancements^30,31^. Similarly, while notable increases in myeloma survival, especially for Non-Hispanic Asians/Pacific Islanders and Non-Hispanic Blacks, suggest effective treatment progress, disparities persist, requiring further investigation^32^. However, the lower survival rates for Non-Hispanic Blacks in NHL compared to other groups indicate a disparity demanding attention^33^.

### Acute leukemia: a concerning trend

The increase in acute leukemia survival is a positive sign of treatment progress. However, the lack of sufficient data and lower survival for Non-Hispanic American Indian/Alaska Native patients in 2015–2019 point to a critical gap in understanding and effectively serving this population^34^.

### Ongoing disparities amidst improvements in cancer survival

Despite notable improvements in cancer survival rates across some racial and ethnic groups^35^, disparities in outcomes remain a significant issue. While certain populations have made gains, these have not been sufficient to close the gap in survival rates, highlighting the ongoing challenge of addressing cancer health disparities^36^. Our analysis indicates that while survival rates for several cancers have improved, persistent disparities between racial and ethnic groups are evident^37^.

For example, Non-Hispanic Black women experienced a 5.2% point increase in breast cancer survival (77.6–82.8%), yet their survival rate remains 9.3% points lower than that of Non-Hispanic White women (90.5–92.1%). In non-Hodgkin lymphoma, Non-Hispanic Black patients saw a substantial 9.3% point increase (57.6–66.9%), but this still leaves a gap of 12% points compared to Non-Hispanic Whites (75.2–78.9%).

For colorectal cancer, survival for Non-Hispanic Black patients rose modestly by 1.5% points (56.8–58.3%), whereas Non-Hispanic American Indian/Alaska Native patients experienced a 6.2% point decline (66.3–60.1%), suggesting widening disparities for this group. The decrease in pancreatic cancer survival for Non-Hispanic American Indian/Alaska Native patients (6.3–3.6%) is troubling, especially when other racial/ethnic groups showed improvement, such as Non-Hispanic Whites (8.4–10.1%) and Non-Hispanic Blacks (5.1–5.7%).

### Implications for healthcare policy and practice

While our analysis did not directly investigate the impact of socioeconomic status (SES), healthcare access, and quality of care on cancer survival disparities, it is essential to acknowledge their potential influence^38^. Numerous studies have demonstrated that these factors significantly contribute to the disparities observed in cancer outcomes^39^. For instance, existing research indicates that lower SES, limited access to advanced medical treatments, and variations in the quality of care received can exacerbate survival disparities among racial and ethnic groups^40^. Although our study focused on survival trends, the broader context of these social determinants should not be overlooked, as they likely play a critical role in shaping the disparities we identified^41^. This aligns with existing literature on the factors influencing cancer survival disparities^35^.

The disparities in cancer survival rates among racial and ethnic groups are likely influenced by social determinants of health (SDOH), such as socioeconomic status, geographic location, and healthcare access, which can delay diagnosis and limit treatment quality. Studies by Shavers and Brown (2002) and Raghavan (2007) emphasize that socioeconomic and healthcare access disparities significantly impact cancer treatment outcomes^4,5^. Recent research by Iyer HS et al. (2023) further highlights that neighborhood-level social environments may have a greater effect on survival rates than genetic ancestry^42^. These findings suggest that SDOH play a critical role in shaping cancer treatment outcomes, influencing healthcare access, treatment adherence, and social support. Future research should explore how SDOH, alongside medical interventions, contribute to the ongoing survival disparities.

These findings underscore the need for targeted interventions to reduce survival disparities^36^. Policy efforts should focus on equitable access to care^43^, addressing systemic barriers, and ensuring advancements in treatment are distributed across all racial and ethnic groups^37^. Tailored strategies in cancer treatment and management, along with expanded healthcare access, are crucial for improving outcomes for all populations^3^.

### Future research and policy directions

Future research should aim to unravel the complex interplay of biological, environmental, socioeconomic, and healthcare system factors contributing to these disparities. Tailoring cancer treatment and management strategies to improve healthcare access is crucial for reducing disparities in health outcomes^1,2,44^.

### Limitations

While our study provided an important update to the seminal work of Pulte et al. (2012), analyzing five-year survival rates across a broad spectrum of cancers and diverse racial/ethnic groups, it came with inherent limitations. Firstly, our methodological framework was deeply rooted in the qualitative approach of Pulte et al., reflecting a continuation rather than an evolution of analysis techniques^1^. Moreover, factors such as socioeconomic status, access to healthcare, genetic predispositions, and treatment differences might have played a role in these disparities. However, the scope of our study was constrained by the unavailability of certain variables, such as socioeconomic status, access to healthcare, and treatment differences, which could not be assessed. These factors, although crucial, were beyond the dataset used, highlighting the need for future research to explore their impact on survival disparities^45–49^. This limitation underscored the need for further research that delved deeper into these factors to fully comprehend and address the causes of survival disparities in cancer treatment outcomes. Additionally, future studies should focus on distinguishing between statistical and clinical significance to better interpret the real-world impact of these results.

## Conclusion

Our analysis highlights progress in cancer survival over the past two decades while revealing ongoing disparities among racial and ethnic groups, particularly in pancreatic and colorectal cancers. The decline in pancreatic cancer survival for Non-Hispanic American Indian/Alaska Native individuals and the overall decrease in colorectal cancer survival were noted. These disparities indicate the importance of evaluating and potentially improving access to advanced cancer treatments and developing targeted approaches to effectively bridge gaps in survival rates across all populations.

## Data Availability

The data that support the findings of this study are available from the SEER database (https://seer.cancer.gov/), but restrictions apply to the availability of these data, which were used under license for the current study, and so are not publicly available. Data are however available from the corresponding authors upon reasonable request and with permission of the SEER database.
